# The policy environment of self-care: a case study of the Philippines

**DOI:** 10.1093/heapol/czac095

**Published:** 2022-11-04

**Authors:** Leonardo Iii Jaminola, Jana Marie Negre, Veincent Christian F Pepito, Arianna Maever Loreche, Manuel M Dayrit

**Affiliations:** School of Medicine and Public Health, Ateneo de Manila University, Ortigas Ave., Pasig City 1604, Philippines; School of Medicine and Public Health, Ateneo de Manila University, Ortigas Ave., Pasig City 1604, Philippines; School of Medicine and Public Health, Ateneo de Manila University, Ortigas Ave., Pasig City 1604, Philippines; School of Medicine and Public Health, Ateneo de Manila University, Ortigas Ave., Pasig City 1604, Philippines; National Clinical Trials and Translation Center, National Institutes of Health, University of the Philippines Manila, Pedro Gil St., Manila 1000, Philippines; School of Medicine and Public Health, Ateneo de Manila University, Ortigas Ave., Pasig City 1604, Philippines

**Keywords:** Self-care, policy review, Philippines, Universal Health Care

## Abstract

Self-care is the ability and empowerment of individuals to maintain health through informed health-care decisions, with or without the support of a health provider. High-income countries have made advances to their conceptualization, research and institutionalization of self-care, given its reported benefits to patients, the health system and economy. A similar undertaking in low- and middle-income countries (LMICs) with already fragile health systems is warranted as highlighted by the coronavirus disease 2019 pandemic. Our article therefore aimed to describe and analyse the policy environment of self-care using the Philippines as a case study, which may have relevance to other similar countries and settings that are transitioning towards Universal Health Care (UHC) to reform and strengthen their primary care systems. We conducted 13 key informant interviews and 2 focus group discussions among representatives from the government, the pharmaceutical retail/industry, community retail pharmacy, primary health physicians and health workers, an infirmary administrator and patients and/or patient advocates. We triangulated our qualitative data with findings from our policy review. We found a total of 13 relevant policies on self-care in the Philippines recently drafted and/or implemented from 2016 to 2021 that fall under the broad categories of unifying frameworks and road maps, capacity building and institutional streamlining, regulations and disease guidelines. Our case study highlights the role of the UHC Law as a driver for self-care and patient empowerment towards better health outcomes with its passage resulting in the promulgation of self-care-related policies. Our findings also suggest that changes in the local policy and built environment, and the formal educational and health systems, are needed to foster a culture of responsible self-care. There are notable exemplars in advancing self-care in the region, including Thailand, from which LMICs like the Philippines can draw lessons to make progress on institutionalizing self-care and, ultimately, realizing UHC and Health For All.

Key messages

- The recent passage of the Universal Health Care (UHC) Law in the Philippines resulted in the promulgation of policies on self-care and served as an organizing and guiding principle, around which more policies on self-care can be created.
- The value of self-care in the World Health Organization South East Asian region in promoting population health and improving health outcomes, particularly in low- and middle-income countries (LMICs), is only being recognized in recent years. A notable exemplar is Thailand in part due to its programmes in capacitating and supporting community health workers, allowing sufficient curriculum time for health, and a taxpayer-funded universal health insurance scheme. The Philippines and other similar LMICs may draw lessons from such models to make progress on institutionalizing self-care and realizing UHC and Health For All.
- We propose the following: (1) developing and institutionalizing a national self-care policy to help guide future self-care interventions; (2) training community health workers and making other investments towards primary care; (3) promoting health literacy and integration of self-care throughout various stages in the life-course; (4) creating information, education and communication materials to guide people towards responsible self-care; (5) providing an environment supportive of responsible self-care with opportunities to pursue self-care activities and (6) expanding mental health and other primary care services in rural and remote communities.

## Introduction

### Self-care: scope, policy and debate

Self-care involves a wide range of choices and practices, shaped by people’s experiences, beliefs and knowledge. There are many definitions of self-care with divergences across disciplines (Vellone *et al.*, 2013; Narasimhan *et al.*, 2019; World Health Organization, 2019a), and the concept of self-care has often been integrated and interchanged with self-efficacy, self-medication, self-management, symptom management and self-monitoring. It has also been associated with responsibility, self-direction and autonomy (Martínez *et al.*, 2021). There are a variety of activities and areas that are considered self-care, which range from daily activities like maintaining personal hygiene and cleanliness in personal spaces to lifestyle and socialization activities such as ensuring proper nutrition and doing physical exercise and sports (Tulu *et al.*, 2021). The World Health Organization (WHO) encapsulated self-care within six areas: (1) promotion of health; (2) prevention and control of disease; (3) self-medication; (4) provision of care to dependent persons; (5) seeking primary, hospital and specialist care if necessary and (6) rehabilitation (World Health Organization, 2019a). It further linked five outcomes with the implementation of self-care interventions: (1) expansion of access and coverage; (2) improvement in the quality of services; (3) higher equity and lower health disparities; (4) better health, social and human rights situation and (5) reduction in cost while having a more efficient utilization of health-care services and resources.

Recognizing the benefits of self-care with billions in cost savings (Global Self-Care Federation, 2021), several high-income countries have formulated policies institutionalizing it. In Scotland and Wales in the United Kingdom, Manitoba in Canada, and some Australian states, policies focusing on self-management of chronic diseases have been implemented. These policies prioritized the provision of self-management skills for chronic disease patients and the conduct of educational programmes for health-care workers (O’Connell *et al.*, 2018). Meanwhile, in England, the Expert Patients Programme—a lay-led self-care support programme—was developed to provide courses that aimed to improve the self-care skills of patients and was viewed as effective in increasing the energy levels and self-efficacy of individuals with long-term conditions (Kennedy *et al.*, 2007). In Ireland, the ‘Living Well with a Chronic Condition: Framework for Self-management Support’ was published in support of an integrated programme to prevent and manage chronic disease with self-management as one of the workstreams (Greaney and Flaherty, 2020). Self-care research and programmes have largely focused on chronic illnesses and noncommunicable diseases (NCDs), and even the proposed research strategy for self-care mirrors this (Riegel *et al.*, 2021). Despite the progress in the field of self-care however, there have also been debates, raising concerns about governmental overreach that encroaches on people’s rights to their personal health and well-being. Self-care policies have also been criticized for their overreliance on patients, especially vulnerable ones, to personal autonomy and responsibility that can result in worse health outcomes. This is exemplified in reports of improper use of medicines for certain conditions, and not consulting with a health-care provider for life-threatening conditions until it is too late.

These advances in the field of self-care have been largely limited to high-income countries, the Americas and Europe, with only one monograph on self-care practices and policies in the WHO South East Asian region published in 2009 (World Health Organization, 2009). The document found that while there are health policies and regulations related to concepts associated with self-care, there are no comprehensive self-care policies in the region and its neighbours, especially among the different low- and middle-income countries (LMICs). The monograph recommended assessing policies to institutionalize self-care; however, no further studies have documented and analysed self-care policies in LMICs since its publication. Fragile health systems in the context of a global health crisis, together with shortages of human resources and limited health-care capacity and health financing (Amit *et al.*, 2021), highlight the need for a nuanced understanding of self-care policies in LMICs. This will enable context-specific recommendations on how to further promote self-care in these countries.

### The Philippine Health System and the need for self-care

The Philippines, an archipelagic LMIC in Asia that is transitioning towards Universal Health Care (UHC), has incrementally improved its health system through legislation. In 1991, the country adopted a devolved model for health governance with the Department of Health (DOH) as the lead implementer of health policies and programmes in the country. This means that the agency is responsible for providing the country with regulatory services, strategic plans and policy directions, and guidelines and standards for health and health care. Aside from these, DOH also manages regional, speciality and government–corporate hospitals (Dayrit *et al.*, 2018). Currently, there are 70 DOH hospitals across the country (Department of Health, 2022a), with both public and private sectors engaged in the delivery of health services in the country. The public sector is operated by the national and local governments and, as such, is mainly funded by taxes. Apart from DOH, local government units (LGU) supervise different types of health facilities and provide various kinds of services. For example, operations of the district and provincial hospitals fall under the function of provincial governments. Meanwhile, municipal governments are responsible for primary care services (e.g. preventive and promotive health services). These services are implemented by rural health units, local health centres and barangay health stations. Municipalities are also in charge of the construction of clinics and health centres and the purchase of medical supplies and medicines (Uchimura, 2012; Dayrit *et al.*, 2018). There are also independent and highly urbanized cities that give services encompassing both primary and hospital care. The private sector, on the other hand, is made up of both for-profit and non-profit organizations providing different kinds of health-care services. Largely market-oriented, health care provided by the private sector is usually paid for by the clients (Dayrit *et al.*, 2018).

The devolution of the health system was then followed by the establishment of a national health insurance programme, the Philippine Health Insurance Corporation (PhilHealth) in 1995 to provide financial risk protection and prevent catastrophic health expenditures (Dayrit *et al.*, 2018). It works by creating benefit packages for certain diseases and procedures, which would be used to subsidize part of the cost of treatment and management and paid directly to the health-care provider upon documentation of service. As of 2021, PhilHealth has >52 million direct and indirect contributors (Philippine Health Insurance Corporation, 2021). However, more than two decades after its establishment, total health spending is still largely out-of-pocket with 52% of health-care expenditures financed by out-of-pocket spending (Department of Health, 2019a), and on average, PhilHealth only covers 30% of health-related expenditures (Dayrit *et al.*, 2018). The burden of paying for health-care services is greater among poorer populations who are more vulnerable to higher health spending in times of critical health situations. They are also more likely to suffer from catastrophic health expenditures, which keep them in poverty (Banaag *et al.*, 2019).

In 2019, what may be the most significant development in the health sector was the passage of the UHC Act (Dumaraos, 2014; Congress of the Philippines, 2019). The law aims to implement a systemic approach to realize universal health in the Philippines and ensure equitable access to quality and affordable health-care services. The law is envisioned to protect Filipinos from financial risk due to health-care expenditures (Congress of the Philippines, 2019). Since its enactment, DOH, together with PhilHealth, has been working on its implementation in both national and local settings and even fast-tracking some developments to aid the country’s response to the coronavirus disease 2019 (COVID-19) pandemic (Department of Health, 2021a). The UHC Law builds on past policies and aligns with the WHO’s definition and concept of self-care. It therefore provides a good platform for self-care, given its emphasis on primary care and health promotion in the population. However, the implementation of the law has been significantly affected by the COVID-19 pandemic.

Through all these reforms, the Philippines made progress in achieving global health targets under the Sustainable Development Goals and its predecessor, the Millennium Development Goals (Dayrit *et al.*, 2018). Even with these developments, challenges for the health sector remain. In terms of health-care capacity, the country only has 1.2 beds per 1000 population (Department of Health, 2020a), which is less than the 2.9 global average of hospital beds per 1000 people as of 2017, or its other neighbours in Southeast Asia (The World Bank, 2020). There have also been issues regarding primary care services in ‘barangays’ (or villages). Only half of the ‘barangays’ in the country are capacitated with at least one barangay health station. Health-care staffing also is a public health issue with only 7.7 doctors per 10 000 population compared with the global average of 15.1 doctors per 10 000 population (World Health Organization, 2019b, 2020). This problem is more pronounced due to the maldistribution of health-care providers, including doctors, throughout the country, with most of them practising in urban areas (Kanchanachitra *et al.*, 2011; Flores *et al.*, 2021; Haakenstad *et al.*, 2022).

Responsible self-care provides a cost-effective way of addressing the shortages and challenges in health-care resources and financing, and advancing the transition to UHC (Amit *et al.*, 2022). The prevalence of self-care among Filipinos has not been previously estimated, but it has been reported that ∼3–6 in 10 Filipinos practise self-medication (Barber *et al.*, 2017), which constitutes an aspect of self-care. If properly implemented and encouraged, the potential of self-care to improve population health and well-being makes it a vital, albeit under-appreciated, tool of a health-care system (World Health Organization, 2009; 2010; Narasimhan *et al.*, 2019).

This article thus aimed to identify and analyse policies on self-care using the Philippines as a case study, with a particular focus on self-care to prevent and manage common acute health conditions including back pain, allergic rhinitis, general acute pain, cough, cold, diarrhoea, constipation and stress. These significantly contribute to the burden of disease and loss of economic productivity (Sanico, 2004; Katelaris *et al.*, 2011; Dutmer *et al.*, 2019; Dierick *et al.*, 2020; Department of Health, 2022b) but can be managed by patients on their own with strengthened health literacy to make responsible and informed health choices.

## Materials and methods

This article used a qualitative case study approach to identify and analyse self-care policies in the country. The findings of this article are part of a larger study on self-care that utilized a mixed methods design to identify local studies and policies on self-care and to assess its social value and economic impact. Our findings are informed by our key informant interviews (KIIs) and focus group discussions (FGDs) on existing self-care policies in the country, conducted from November 2021 to February 2022, and triangulated by our policy review conducted from January 2022 to March 2022. The policy review followed the general steps of Joanna Briggs Methodology (Peter *et al.*, 2020; Antonio *et al*., 2021). We reported the findings of our KIIs, FGDs and policy review following the consolidated criteria for reporting qualitative research (Tong *et al.*, 2007) and Preferred Reporting Items for Systematic Reviews and Meta-Analyses Extension for Scoping Reviews (Tricco *et al.*, 2018) guidelines, respectively. Further information is provided in the Supplementary Appendix.

## Results

### Search results

**Figure 1.**
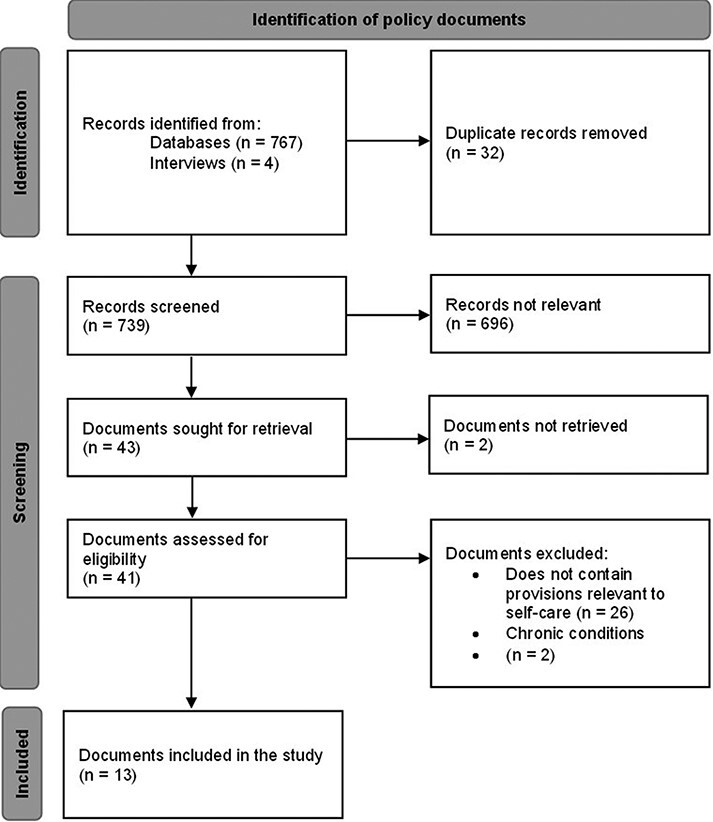
Policy review diagram

There were 767 records identified from the three databases and four records from expert interviews, with a total of 32 duplicate records. Based on the titles of the records, only 43 were deemed relevant while the remaining 696 were excluded. Two records were not accessible. Of the 41, a total of 26 records did not contain provisions relevant to self-care while two focused on chronic conditions. Our study therefore includes data from 13 policy documents (Figure 1).

### Summary of findings

All 13 policy documents identified through the interviews, FGDs and policy review were only institutionalized from 2016 onwards, with 12 of them only institutionalized from 2018 onwards (House of Representatives, 2016; 2019; 2021; Congress of the Philippines, 2018; 2019; Food and Drug Administration, 2019; Department of Health, 2019b,c; 2020b,c; 2021b,c,d) (Table 1). Six documents were from the DOH Document Management and Archiving System database, five were from the House of Representatives Legislative Information System database with one also found in the Senate database and two were only identified through the interviews [i.e. one from DOH and another from the Food and Drug Administration (FDA)]. Two of the policy documents are laws, seven are DOH Administrative Orders (AOs) or Circulars, one is an advisory from the FDA and three are house bills.

**Table 1.** Summary of policy documents related to self-care

Type	Subject/title	Year	Short summary of the document	Source
Law	Republic Act 11036: An Act Establishing a National Mental Health Policy for the Purpose of Enhancing the Delivery of Integrated Mental Health Services, Promoting and Protecting the Rights of Persons Utilizing Psychiatric, Neurologic and Psychosocial Health Services, Appropriating Funds Therefor, and for Other Purposes	2018	The law aims to strengthen leadership and governance for mental health, develop a comprehensive, integrated, effective and efficient national mental health-care system, protect the rights and freedoms of persons with mental health needs, strengthen information systems, evidence and research, integrate mental health services into the basic health services and integrate the promotion of mental health into educational institutions, the workplace and the community.	Congress of the Philippines (2018)
Law	Republic Act 11223: An Act Instituting Universal Health Care for All Filipinos, Prescribing Reforms in the Health Care System, and Appropriating Funds Therefor	2019	The UHC Act of 2019 was created to uphold the policy of the State to protect and promote the right to health of the Filipino people. It aims to realize UHC through a systemic approach and clear delineation of roles among the key agencies and stakeholders. It also aims to ensure equitable access to quality and affordable health-care goods and services, especially emphasizing the NHIP to protect each Filipino against financial risk.	Congress of the Philippines (2018)
DOH AOs and Circulars	Adoption of the National Food and Waterborne Disease Prevention and Control Program Clinical Practice Guidelines on Acute Infectious Diarrhoea Reference Manual	2019	The circular announced the adoption of the CPG on Acute Infectious Diarrhoea Reference Manual to strengthen the implementation of the FWBD Prevention and Control Program.	Department of Health (2019b)
DOH AOs and Circulars	Implementing Rules and Regulations of the Universal Health Care Act (RA 112233)	2019	This document outlines the IRR of the UHC Act. It covers all the sections also mentioned in the UHC Act and provides technical guidance to the key agencies and stakeholders concerned with the implementation of the specified law.	Department of Health (2019c)
DOH AOs and Circulars	Health Promotion Framework Strategy in Province-wide and City-wide Health Systems	2020	The order aimed to provide guidance and direction for P/CWHSs to operationalize health promotion as envisioned in the UHC Law.	Department of Health (2020b)
DOH AOs and Circulars	Guidelines on the Transformation of the Health Promotion and Communication Service (HPCS) to the Health Promotion Bureau (HPB)	2020	This order aimed to provide guidance on the transformation of the HPCS to a full-fledged HPB by virtue of the UHC Act.	Department of Health (2020c)
DOH AOs and Circulars	Health Promotion Framework Strategy 2030	2021	The HPFS serves as the national health promotion road map and the basis for all policies, programmes, plans and activities on health promotion. The HPFS provides foundational strategies for (1) increasing health literacy with a focus on reducing NCDs, (2) implementing population-wide health promotion interventions across social determinants of health, (3) exercising policy coordination across government instrumentalities to ensure attainment of the framework strategy and its programmes and (4) providing technical support to local research and development relevant to the directions of the HPFS.	Department of Health (2021b)
DOH AOs and Circulars	Guidelines on the Implementation of Participatory Action Research for Health Promotion and Social Mobilization Pursuant to Republic Act No. 11223	2021	The order provided the overall framework and guidelines for the operationalization of PAR for health promotion and social mobilization.	Department of Health (2021c)
DOH AOs and Circulars	Public Consultation on the Draft DOH Administrative Order entitled ‘Guidelines for Implementing Participatory Action Research for Health Promotion and Social Mobilization Pursuant to Republic Act No. 11223, Establishing the Realizing Equity through Sustainable Health Action, Participation and Empowerment (RESHAPE) Program Thereof’	2021	A public consultation on the draft of the DOH for guidelines for implementing PAR is disseminated to gather comments from its stakeholders prior to the release of the guidelines. The main mechanism to operationalize PAR is through the RESHAPE Programme. The implementation of PAR is mandated by the UHC Act to focus on cost-effective and high-impact interventions for health promotion and social mobilization.	Department of Health (2021d)
FDA Advisory	Internet Sales and Access to Safe Medicines	2019	Due to the growing online market, including the sale of medical products, the FDA advises the general public that the current law does not permit the online selling of medicines. Only online ordering services of existing FDA-licensed pharmacies with a physical address may be permitted.	Food and Drug Administration (2019)
House Bill	House Bill 03657: An Act Institutionalizing Health Promotion in the Philippines by Establishing a Philippine Center for Health Promotion and Disease Prevention, Providing for a Health Promotion Fund and for Other Purposes	2016	The Act aims to mainstream health through population-based approaches. To this end, a Philippine Center for Health Promotion and Disease Prevention is created as a corporate body. Thus, it is mandated to have corporate powers such as ownership of property and engagement in contracts. Furthermore, a Health Promotion Fund from the incremental revenues allocated for health is established to fund the operations and activities of the Philippine Center for Health Promotion and Disease Prevention.	House of Representatives (2016)
House Bill	House Bill 5515: An Act Establishing a Health Promotion Fund and Health Promotion Commission to Oversee the Implementation of Health Promotion in the Philippines and for Other Purposes	2019	The policy seeks for people to increase control over their health and its determinants through a comprehensive and coordinated approach for health promotion. The purposes of the bill are the following: mainstream health in public policy and among various sectors, establish a Health Promotion Commission and establish a Health Promotion Fund. The Health Promotion Commission is to be an independent, autonomous body with the same status as a national government agency. Meanwhile, the Health Promotion Fund will be sourced from 20% of the remaining incremental revenues allocated for health.	House of Representatives (2019)
House Bill	House Bill 10059: An Act Strengthening the Traditional and Complementary Medicine System, Amending for the Purpose Republic Act No. 8423, Otherwise Known as the ‘Traditional and Alternative Medicine Act of 1997’	2021	Following the Traditional and Alternative Medicine Act of 1997, this bill seeks to modify and strengthen its provisions, especially regarding the regulatory and enforcement of regulations. The purpose of the bill is to improve the quality and delivery of health-care services through the development and proper regulation of traditional and alternative health care.	House of Representatives (2021)

### Themes

Policies did not use the term ‘self-care’ but described self-care-related interventions and concepts including health promotion and health literacy. From these policy documents, we thematically identified four key themes: (1) unifying frameworks and road maps, (2) capacity building and institutional streamlining, (3) regulations and (4) disease guidelines.

The first theme, unifying frameworks and road maps, covers fundamental laws and policies that set goals and frameworks regarding health and the health-care system in the country. These cover republic acts and policies on health promotion and self-care strategies and serve as a basis for future and more specific policies and implementation guidelines. Meanwhile, documents classified under the theme of capacity building and institutional streamlining aim to establish and delineate the role of different agencies and offices in the implementation of health-related policies and strengthen the capacity of the health system on health promotion and self-care. Documents on the control of sale, access and distribution of medicines and health products and services fall under the theme of regulations. Lastly, disease guidelines refer to documents dedicated to the management of a particular disease with a focus on suggested self-care interventions.

#### Unifying frameworks and road maps

The UHC Act of 2019 and its Implementing Rules and Regulations (IRR) are crucial national policies in the formation of a healthier population. Under the UHC, the envisioned framework adopts a whole-of-society approach in health protection and promotion. Moreover, the UHC Law seeks to refocus the efforts of the health-care system towards primary health care indicating a focus on self-care practices. This entails encouraging individuals towards healthy living and protection from health risks and empowering them through health literacy (Table 2, Quote U1).

**Table 2.** Illustrative quotes

Code	Theme	Illustrative quote
U1	Unifying frameworks and road maps	‘UHC is the blueprint for the health system and health-care delivery system reforms, with some provisions that emphasize self-care. If you look at the Principles and Policies, the UHC protects and promotes the right to health of all Filipinos and instils health consciousness. Another part of UHC is in contracting networks, where it implements and integrates systems into provider networks. It is written in the provisions that the primary care network is the foundation of the health-care delivery system. The primary care network shall provide primary care services, serving as initial contact and navigator to guide patients’ decision-making for cost-efficient and appropriate levels of care. Patients’ decision-making on their health is encouraged, which is only possible if they have proper information, a health-care delivery system and policy in place. The UHC Law is the basic policy and regulation for self-care.’ (Participant 4, KII with a government representative)
U2	Unifying frameworks and road maps	‘Under the UHC Law, PhilHealth should be implementing a primary case benefit package like Konsulta, If patients are empowered, or if patients do self-care, whether seeking consultation at an early stage or whether treating themselves through herbals and other cultural practices of addressing diseases, and it is effective—then it will lessen the government’s expenses on catastrophic illnesses.’ (Participant 4, KII with a government representative)
C1	Capacity building and institutional streamlining	‘We have playbooks that support the behaviours in the [HPFS] priority areas, which the LGUs can easily work on. If we think that getting people to ride bikes, doing active transport is a mode of self-care, then the playbook teaches the LGUs how to build bike lanes, for example, in their locality. If we say self-care is eating healthy, we have a playbook on how “carinderias” [or food stalls] can serve healthier food.’ (Participant 7, KII with a government representative)
P1	Policy recommendations	‘Self-care should be integrated into the academic curriculum so that students know how and when to do self-care at an early age. Self-care should also be promoted at the workplace, together with campaigns of LGUs to raise awareness and promote self-care. The general public should then be reminded through infomercials and infographics. Together with encouraging a culture of self-care, we also need to encourage a culture of research and fact-checking for reliable resources. And finally, the built environment should be enabling of self-care. This means having parks, open spaces, libraries and avenues for individuals to safely engage in self-care activities that improve health. All this is important for the public to know that self-care and resources that support it are available, especially among Filipinos who are resistant to ideas and changes that they are unfamiliar with. If they perceive it to be within their capacity, then they might engage in responsible self-care more.’ (Participant 6, KII with a government representative)
P2	Policy recommendations	‘Our voice needs to be institutionalized in our regulatory and policy environment. Most of the time, the government crafts policies and uses consultations as a sounding board—which is not ideal. The ideal situation is, we [patients] are part of the creative process, the brainstorming and analysis, and not just asked to comment on the final draft policy.’ (Participant 1, FGD among patients and/or patient advocates)
P3	Policy recommendations	‘There needs to be co-ownership of health policy even for non-health agencies. There is a need for non-health agencies to fully support these policies as if it is their mandate as well. We have technical working groups, but people still recognize it as the primary work of the DOH. Even schools need to work towards the agenda for them.’ (Participant 7, KII with a government representative)

As part of the law, the National Health Insurance Program (NHIP) was also expanded to protect Filipinos from financial difficulties during medical situations and ensure the provision of health care for all. For this purpose, every citizen is to be automatically included in the NHIP for immediate access and eligibility in availing of health services. The most recent primary care package by PhilHealth is called the ‘Konsultasyong Sulit at Tama’ (‘Konsulta’) (Table 2, Quote U2).

Despite this, Filipinos do not go ‘even if there is free testing, diagnostic and treatment, unlike when there is a shoe sale at 50% and you see them start lining up in the morning. Health is their last priority’ (Participant 5, FGD among patients and patient advocates). Beyond affordability, services need to be ‘available, accessible and convenient to access. Especially those who are working, they should be able to access services even on weekends or even 24/7. There is no shortage of innovation. We can definitely drive behaviour change if we address patient needs and preferences’ (Participant 1, FGD among patients and patient advocates).

In line with the UHC and recognition that health literacy among Filipinos needs to be strengthened, the Health Promotion Framework Strategy (HPFS) was developed. The DOH AO 2021-0063 (‘Health Promotion Framework Strategy 2030’) further substantiated health promotion provisions in the UHC Law. The framework serves as the foundation for the development and nationwide implementation of health promotion policies, programmes, plans and activities. The HPFS enumerates seven priority areas for health promotion: diet and physical activity, environmental health, immunization, substance abuse, mental health, sexual and reproductive health, and violence and injury prevention. Using the Ottawa Charter and Bangkok Declaration for Health Promotion as a reference, the HPFS identified five action areas for implementation: developing healthy public policies, creating supportive environments, developing personal skills, strengthening community action and reorienting health services. While all these areas are related to self-care, the most relevant are the development of personal skills and the reorientation of health services. The development of personal skills entails the provision of applicable and useful knowledge to allow for the adoption of health-seeking behaviours. Meanwhile, the reorientation of health services calls for shifting focus on disease prevention and health promotion which includes strengthening self-care practices in the country. The framework also prescribes the use of health literacy as a key strategy to further the promotion of health. This particular strategy necessitates interventions to improve the ability to process, understand and apply health-related information, encourage health-seeking behaviours and practices and apprise decisions related to health. This is a crucial element in the adoption of self-care, given that it is influenced by an individual’s level of health literacy.

Finally, Republic Act No. 11036 or the Mental Health Act of 2018 is another key legislation containing provisions relating to health promotion and self-care that was identified in our qualitative study: ‘I know that the Mental Health Law is meant to ensure that services are provided to individuals with regards mental health issues, which in a way impacts self-care’ (Participant 1, KII with a primary care physician). The law focuses on the integration of comprehensive and effective mental health care not only into the health-care delivery system of the country but also into educational institutions and the workplace. Specific self-care provisions contained in the law include strengthening public awareness campaigns on mental health issues and mental health promotion in educational institutions and the workplace. Moreover, the law also notes the provision of home care services for patients with special needs concretizing the role of self-care in mental health treatment.

#### Capacity building and institutional streamlining

The passage of the UHC Act paved the way for several developments in the DOH in relation to capacity building and institutional streamlining. As a response to UHC’s directive on the transformation of the Health Promotion and Communication Services (HPCS) to the Health Promotion Bureau (HPB), DOH released AO 2020-0058 (‘Guidelines on the Transformation of the Health Promotion and Communication Service (HPCS) to the Health Promotion Bureau (HPB)’) specifying and clarifying the mandates, roles and responsibilities of the HPB as a branch of the DOH. Under this, the HPB was tasked to coordinate with other concerned bureaus and agencies in the advancement of health promotion. Strategies and programmes of the HPB are expected to address the following: (1) behavioural risk factors, (2) social determinants of health, (3) functional health literacy and (4) healthy settings. Aside from this, there have also been efforts to localize health promotion initiatives through AO 2020-0042 (‘Health Promotion Framework Strategy in Province-wide and City-wide Health Systems’) mandating the implementation of health promotion in province-wide and city-wide health systems (P/CWHSs). With the HPFS in P/CWHSs, the DOH designed and implemented programmes related to it (Table 2, Quote C1).

The document also provided guidance and direction for LGUs to operationalize the health promotion mandate stipulated under the UHC and HPFS. The AO also defined the local governance structure of health promotion programmes designating the Provincial/City Health Board as the lead in implementing and evaluating health promotion programmes and policies in the P/CWHS. Through its LGUs, P/CWHSs are tasked to implement health promotion measures including self-care practices such as physical activity, proper nutrition and personal hygiene. The emphasis of policies on preventive aspects, especially among NCDs, was also noted by our participants: ‘I am not aware of any laws on self-care. At the local level, LGU policies are focused on promotion and prevention of NCDs’ (Participant 4, FGD among primary care physicians).

In addition, two records referred to the inclusion of Participatory Action Research (PAR) for health promotion and social mobilization in the national research agenda. DOH formulated a draft policy creating the Realizing Equity through Sustainable Health Action, Participation and Empowerment (RESHAPE) Programme to operationalize provisions regarding PAR in the UHC. The draft policy aims to establish guidelines regarding the provision of financial and technical assistance to implement PAR projects, produce PAR-trained health practitioners and make a network of researchers and institutions that can employ PAR methodology. In relation to this, DOH released Department Circular 2021-0456 (‘Public Consultation on the Draft DOH Administrative Order entitled “Guidelines for Implementing Participatory Action Research for Health Promotion and Social Mobilization Pursuant to Republic Act No. 11223, Establishing the Realizing Equity through Sustainable Health Action, Participation and Empowerment (RESHAPE) Program Thereof”’) to solicit comments on the draft policy. This was followed by the publication of AO 2021-0065 (‘Guidelines on the Implementation of Participatory Action Research for Health Promotion and Social Mobilization Pursuant to Republic Act No. 11223’). The guidelines enumerated themes to be prioritized in PAR projects, which are aligned with the same seven priority areas listed in the HPFS. Through this, the studies of healthy behaviours and self-care are pushed into the national research agenda. The focus on PAR is expected to yield a better understanding of the determinants of health relevant to different communities, examine the linkages between health system performance and social roles and responsibilities and cover the gap between knowledge and practice. As it is focused on health promotion, better self-care interventions for different medical conditions suitable in various contexts can be developed.

Our case study also found two bills that proposed the creation of a central agency for health promotion and a fund dedicated to the works of the agency: House Bill 3657, or the Health Promotion and Disease Prevention Act, and House Bill 05515, or the Health Promotion Act of 2019. The former was filed in September 2016, while the latter was filed in November 2019 (or after the enactment of the UHC Law). In general, these bills cover similar scopes but differ in their approaches and proposed implementation, such that our study is treated as different bills. Both bills seek to allocate funding sourced from taxes collected from the purchase of alcohol and tobacco for the use of the health promotion agency. However, House Bill 3657 proposed that the Philippine Center for Health Promotion and Disease Prevention be organized as a corporate body guided by a Board of Trustees. Meanwhile, House Bill 05515 forwarded the creation of the Health Promotion Commission, a national government agency, as the central body for health promotion. In the bill, the commission is set to work with other government agencies such as the Department of Agriculture, the Department of Education and the Department of Public Works and Highways (DPWH), among others to mainstream health in various areas. For example, the DPWH, together with the Metro Manila Development Authority, is called to design plans which ensure road safety. Moreso, parks and bike lanes are specified as a means of encouraging physical activities and mental wellness. Despite the differences, the ultimate goal of these proposals is to establish an agency to lead the health promotion efforts of the government, mainstream health in all policies and guide the population towards positive health behaviours. Presently, the responsibility is undertaken by the HPB as stipulated in the UHC Act.

#### Regulations

We identified two regulations: House Bill 10059 (‘An Act Strengthening the Traditional and Complementary Medicine System, Amending for the Purpose Republic Act No. 8423, Otherwise Known as the “Traditional and Alternative Medicine Act of 1997”’) and Advisory 2019-154 (‘Internet Sales and Access To Safe Medicine’) issued by the country’s FDA. WHO lists self-management, including self-medication, as one of the areas of self-care (World Health Organization, 2019a). Hence, the regulation of accessible self-care interventions such as medicinal products is regarded as a crucial element in bolstering self-practices.

House Bill 10059 aims to update Republic Act 8423 or the Traditional and Complementary Medicine Act of 1997 and modify provisions related to regulation and its enforcement by boosting the powers and functions of the Philippine Institute of Traditional and Alternative Healthcare (PITAHC). Specifically, the bill mandates PITAHC to ensure compliance of traditional, complementary and alternative medicine (TCAM) facilities with rules and regulations, grant licences for the practice of TCAM and develop product standards and requirements for TCAM products. The development and implementation of regulations on traditional and alternative health care are argued to lead to better quality and delivery of health-care services. However, there are perceptions that ‘over-regulating products discourage adoption’ (Participant 5, KII with a pharmaceutical retail/industry representative).

Aside from this, Advisory No. 2019-154 issued by the FDA in 2019 tackles the sale of medical products online as a response to the increasing sale of medicines online. The advisory was released to serve as a reminder to the general public that current laws do not permit the online selling of medicines. This problem is not limited to the Philippines as it has also been experienced in other countries and has even been regarded as a global phenomenon (Vida *et al.*, 2020). Beyond conventional products like over-the-counter medicines being sold online, the sale of traditional therapies such as Chinese herbal products has also been on the rise on the internet (He and Shi, 2021).

#### Disease guidelines

The Philippines has few policy guidelines providing step-by-step instructions on how to treat certain diseases at home or by oneself. In this study, DOH Circular 2019-0233 (‘Adoption of the National Food and Waterborne Disease Prevention and Control Program Clinical Practice Guidelines on Acute Infectious Diarrhea Reference Manual’) was the only document found related to the treatment of acute diseases. Through this, the Clinical Practice Guidelines (CPG) on Acute Infectious Diarrhoea Reference Manual to strengthen the implementation of the National Food and Waterborne Disease (FWBD) Prevention and Control Program was officially adopted. The aim of the guidelines is to standardize the approach regarding the diagnosis, management and prevention of acute infectious diarrhoea. The guidelines recommended several home interventions for mild cases of diarrhoea. For example, the formula for homemade oral rehydration solution (ORS) was described. The guidelines further noted that previously healthy adults who contract acute diarrhoea may also be managed at home with adequate ORS intake, approximately two times the estimated gastrointestinal losses. For health-care workers, there are also detailed criteria provided under what conditions can an adult with diarrhoea be sent home for treatment. Several of our participants in the KIIs and FGDs mentioned taking ORS, probiotics and over-the-counter medicines to self-manage diarrhoea: ‘When I have diarrhoea, I just hydrate myself. If it is bad, I mix sugar and salt in water and drink that solution’ (Participant 4, KII with a government representative). Non-traditional treatments were also identified: ‘In our communities, there are many concoctions to manage diarrhoea including banaba or avocado leaves’ (Participant 2, KII with a patient and/or patient advocate).

#### Policy recommendations

Policy recommendations to promote self-care in the Philippines varied greatly between the different stakeholders. But there was consensus that changes in the local policy and built environment, and the formal educational and health systems, are needed to foster a culture of responsible self-care. A central theme in our case study is the importance of strengthening health literacy, promoting self-care among children and young adults and having a built environment supportive of self-care (Table 2, Quote P1).

From the perspective of patients and patient advocates, they highlighted the need for patient and community involvement in policy-making (Table 2, Quote P2).

A participant also mentioned the importance of ‘providing routine and outpatient mental health services in rural areas’ (Participant 9, KII with a patient and/or patient advocate), highlighting the gaps between UHC ambition of providing health care to all and the inequities especially in remote and rural communities. A representative from the pharmaceutical sector suggested ‘making medications more easily accessible to people, but also having guidelines to prevent abuse’ (Participant 8, KII with pharmaceutical retail/industry representative). While DOH is leading the implementation of health programmes and initiatives, other government agencies and departments should also support the health agenda (Table 2, Quote P3).

Participants were unanimous in saying that how these policies are implemented also matters. One representative (Participant 3) from the pharmaceutical retail/industry said that ‘health promotion should be done at the local government and even the barangay level’, and an infirmary administrator (Participant 13) mentioned that individuals may be more receptive if health promotion is done on a person-to-person basis.

## Discussion

The UHC Law paved the way for self-care and patient empowerment in the Philippines through legislation, with the majority of the policy documents drafted and/or institutionalized during and after its passage in 2019. The recent development and institutionalization of self-care-related policies in the Philippines highlight the infancy of a formal and unified conceptualization of self-care relative to its advanced development in high-income countries, which may be the experience of other similar LMICs as well. In a 2009 monograph, member countries of the WHO South East Asian Regional Office described self-care policies as being focused on specific aspects of self-care such as TCAM and health promotion (World Health Organization, 2009), similar to the existing Philippine policies. Notably absent in all countries assessed is a national self-care policy; however, among the countries in the monograph, Thailand was significantly advanced with its numerous healthy public policies. This is corroborated in the 2021 Self-Care Readiness Index Report (Global Self-Care Federation, 2021), which assigns a high Self-Care Health Policy score to the country, even higher than developed countries like France, the UK and the USA. The report attributes Thailand’s performance to its tax-funded universal health insurance implemented since 2002, a system that allows its health-care providers to bill for time spent discussing self-care with patients and regulates the practice of TCAM and a strong primary care system including a successful Village Health Volunteer Program. This programme trained local leaders to build trusting relationships, recognize certain conditions, prevent NCDs, serve as early warning and disease surveillance systems and provide basic consultations. Together with sufficient time allotted for physical and mental health throughout the basic education curriculum, this programme was hailed as the main reason for improving health literacy at the community level, especially during the COVID-19 pandemic (Global Self-Care Federation, 2021). In contrast, there are no existing mechanisms by which self-care expenses are reimbursed in the Philippines. The most recent primary care package of the Philippine, the Konsulta package, was identified in our qualitative data. It is a benefit package that aims to encourage access to primary care by reducing outpatient expenditures for routine primary care services and medicines (Philippine Health Insurance Corporation, 2020). While mentioned, we did not include it in the policy documents of our case study since it focuses more on promoting access to primary care within the formal health system, rather than reimbursing self-care expenses. The Philippines also has similar volunteer health workers to Thailand—the ‘barangay’ (or village) health workers (BHWs)—who have been essential in the workforce and health programmes (Mallari *et al.*, 2020). However, as a result of decentralization of health-care delivery, the effectiveness and functioning of BHWs vary depending on the resources and politics of a locality (Dodd *et al.*, 2021). Unlike Thailand, the Philippine primary care system needs to be strengthened and its health workforce well supported and financed. With investments in self-care interventions, there should also be enough curriculum time for health as well as investments in community health workers, such as in the provision of technical training and incentives, among others, as this will improve health literacy and empower people to do self-care (Mallari *et al.*, 2020; Global Self-Care Federation, 2021). While Thailand is an exemplar LMIC in the promotion of self-care, the report noted that one indicator Thailand may have to work on is the recognition of the economic value of self-care (Global Self-Care Federation, 2021), which necessitates the evaluation of the impact of of self-care policies to inform government decision-making. Similarly, the Philippines should also fund research on the economic impact and value of self-care in the country.

A continuing debate especially among countries with advanced TCAM regulation, which is one aspect of self-care, is its integration into the formal health-care system due to its perceived lack of effectiveness and safety (Lee Mendoza, 2009). However, misconceptions on regulation and over-regulation of such products remain among individuals who perceive them to be safe (Marinac *et al.*, 2007), despite concerns by regulatory bodies about their safety and effectiveness. This debate and lack of consensus on TCAM use may be why the Philippines only has a single policy on strengthening TCAM in the country between 2010 and 2022. The purpose of House Bill 10059 was to amend Republic Act 8423 or the Traditional and Alternative Medicine Act of 1997, which has a 7-fold aim: (1) encourage scientific research on TCAM, (2) promote and advocate the use of TCAM, (3) develop and coordinate skills training courses for TCAM modalities, (4) formulate standards for practice of TCAM and manufacture of natural and organic products, (5) formulate policies for protection of indigenous and natural health resources, (6) formulate policies to strengthen the role of TCAM in the health system and (7) promote traditional and alternative health care in different venues. The 1997 law also established PITAHC which has supported studies on drug development from natural products (Congress of the Philippines, 1997). However, since then, no TCAM bill has been passed into law, and a strong TCAM regulation is still lacking in the country. Similarly, most of the published TCAM studies in the country still focused on drug development studies and studies on the behavioural aspects of TCAM use (Palileo-Villanueva *et al.*, 2022). The bill strengthening TCAM in Congress could be improved by legislating stricter regulation of TCAM modalities in the market, encouragement of research on TCAM–drug interactions and encouragement of health information literacy on TCAM and self-medication.

The COVID-19 pandemic has highlighted the need to empower patients and decrease the burden on the health system. It further served as a catalyst for the mainstreaming of health promotion in the country. Of note are recent Philippine House Bills on health promotion, which put greater emphasis on its implementation in local governments, allowing the implementation of health promotion activities applied to local needs and contexts (Department of Health, 2020b; 2021b). It remains to be seen if these House Bills are eventually passed into law with the requisite appropriations. In addition to having enabling laws for self-care, our findings suggest that it is critical to improve health literacy or the ability to identify, understand, evaluate and apply information and services to make health-related decisions such as understanding prescription drug instructions and navigating the health system (US Department of Health and Human Services, 2021). Previous studies illustrated the link between low levels of functional health literacy and poorer health outcomes (Gazmararian *et al.*, 2003; Adams, 2010). The health literacy–health nexus has been well studied among chronic disease patients wherein adequate health literacy was seen to improve outcomes of self-management among asthma, diabetes and even cancer patients (Papadakos *et al.*, 2018). Our case study similarly found a greater focus on noncommunicable and chronic diseases and general health compared with self-limiting conditions. These conditions substantially contribute to disease burden, can be managed with proper guidance and should therefore be included in policies and the proposed research agenda on self-care (Riegel *et al.*, 2021). Given that research and policy should place the needs of individuals and communities at the centre, PAR as advocated by DOH Circulars and AOs should be considered as a design and methodology. Finally, as with TCAM, over-the-counter medications that are commonly used to manage acute conditions should be better regulated to avoid irrational use and antimicrobial resistance (Rather *et al.*, 2017).

The establishment of a National Self-Care Service with the general aim of embedding self-care in the provision of routine care and engaging different stakeholders to practice self-care for better health (Nichols *et al.*, 2020) is one policy that the Philippines can adopt. The integration of self-care in the continuum of primary care is done by enabling patients, consumers, laypeople and the media to be active partners in health care, maintaining quality, regular quality assurance and monitoring and evaluation. Our findings suggest that different stakeholders, agencies, implementers and patients need to be involved to support responsible self-care. This may be a tall order for LMICs, but studies in Brazil show that for every dollar invested in self-care activities, at least six dollars of health-care expenditures are prevented (Global Self-Care Federation, 2021). Analyses from the USA have shown that investments in self-care save billions in health-care expenses and hours for both patients and providers, improving productivity and efficiency (Global Self-Care Federation, 2021). Given the challenges and gaps in the Philippine health system and resources, self-care is a cost-effective strategy towards the realization of UHC and Health For All.

## Conclusions

There is progress towards conceptualizing and/or integrating self-care into the health systems of LMICs, as illustrated by the Philippine case study. In the country, we found that 13 policies were only recently drafted and/or institutionalized during and after the passage of the UHC Act of 2019. Among these, four policy documents were categorized under unifying frameworks and road maps, six were categorized under the capacity building and institutional streamlining theme, two were categorized as regulations and one was categorized under disease guidelines. Among the 13 records, six stemmed from the passing of the UHC Act, highlighting the singular importance of the UHC Act in institutionalizing self-care and strengthening health promotion strategies in the Philippines. Moreover, the UHC Act and these policies associated with the UHC represent the growing importance of self-care in realizing Health For All and transitioning the Philippine health-care system from a curative health system to a primary health system. The COVID-19 pandemic hastened the mainstreaming of health promotion initiatives in the country, and while these recent policies and administrative issuances are a start, much more needs to be done to improve people’s understanding about self-care within the context of primary health care to achieve the goal of enhancing the health and quality of life of citizens. Additionally, self-care can be enhanced if we have strong institutions that provide information and confidence to individuals to take care of themselves. Previous analyses have shown the value of self-care and having a comprehensive national self-care strategy, which benefits not only the economy and health systems but patients and providers as well. We identified Thailand as an exemplar, which has outperformed high-income countries in terms of health literacy and self-care policies and outcomes. Other LMICs can draw lessons from such models and experiences to further improve their policies on self-care. Finally, we propose the integration of self-care into the educational and health-care systems, taking into account the evidence and critiques from other countries, which will be even more vital as LMICs accelerate progress on UHC.

## Supplementary Material

czac095_Supp

## Data Availability

The data underlying this article are available in the paper, with policy documents accessible through the links provided in our reference list.

## Reflexivity statement

L.J., J.M.N., A.M.L. and V.C.F.P. are research specialists in the fields of medicine, health policy and public health. M.M.D. is a health systems researcher and former policymaker. He is a Former Secretary of Health of the Republic of the Philippines and Director of the Department of Human Resources for Health at the WHO. All researchers are based in the Philippines.
