# Supplementary material for: The policy environment of self-care: a case study of the Philippines
Source: Health Policy Plan. 2022 Nov 4;38(2):205–17. doi: 10.1093/heapol/czac095 (PMC9923374; doi:10.1093/heapol/czac095)
Supplement: czac095_Supp [file czac095_supp.zip › Appendix.docx]

**Appendix**

**Appendix A. Detailed description of materials and methods**

**Key informant interviews (KIIs) and focus group discussions (FGDs)**

***Research team and reflexivity***

LJ, JMN, AMLA, and VCFP are research specialists in the fields of medicine, health policy, and public health. MMD is a health systems researcher and former policymaker. He is a Former Secretary of Health of the Republic of the Philippines and Director of the Department of Human Resources for Health at the World Health Organization. All researchers are based in the Philippines.

***Design and participant selection***

We conducted 13 online KIIS and two FGDs. Our key informants were representatives from the government (n=3; Department of Health, Philippine Health Insurance Corp., Food and Drug Administration), the pharmaceutical retail and industry (n=3), and community retail pharmacy (n=2). We also interviewed one health worker, an infirmary administrator in a rural setting, and three patients and/or patient advocates. Additionally, we conducted two FGDs – one among five primary care physicians and another among six patients and/or patient advocates. Participants were selected based on their years of experience and expertise (i.e., government representatives, pharmaceutical retail/industry, health workers). For patients and patient advocates, both urban and rural settings were represented (**Supplementary Appendix Table S1**). The varied representation in our qualitative study allowed us to capture as many possible perspectives as possible on self-care and self-care policies. All participants whom we contacted, with the exception of one invited individual, completed the interviews.

**Supplementary Table 1. Distribution of participants according to classification and setting (n=24)**

| **Classification** | **KII** | **FGD** |
| --- | --- | --- |
| Department of Health | 1 |  |
| Philippine Health Insurance Corp. | 1 |  |
| Food and Drug Administration | 1 |  |
| Pharmaceutical retail/industry | 3 |  |
| Community retail pharmacy  Urban  Rural | 2 |  |
| Facility administrator  Urban  Rural | 1 |  |
| Primary care physician  Urban  Rural | 1 | 5 |
| Patient and/or patient advocate  Urban  Rural | 3 | 6 |
| **Total** | **13** | **11** |

***Instruments and data collection procedures***

We conducted a total of 13 interviews and two FGDs from November 2021 to February 2022. Data collection was conducted via online platforms such as Zoom or via phone call, and face-to-face where possible considering government restrictions imposed during the COVID-19 pandemic. We recruited participants until saturation was reached (i.e., no new information was being obtained from additional interviews and FGDs). The interviews lasted between 60 to 120 minutes with a token amounting to USD 26 (1 USD = 57 PhP as of September 21, 2022) provided to each participant. All participants consented to the interview being recorded.

To elicit responses on self-care and policies, we provided a total of eight commonly encountered scenarios related to the acute conditions of interest: low back pain, diarrhea, colds and cough, allergic rhinitis, general acute pain, constipation, and stress. We then asked their insights and experiences on self-care, which ended with the following policy questions: (1) What are the policies and regulations governing self-care; (2) What is the relevance of self-care to UHC implementation; and (3) What are your policy recommendations to encourage self-care in the Philippines.

***Data analysis and reporting***

The interviews were digitally recorded, transcribed verbatim, and translated from Filipino or Cebuano to English. The research team are native and/or fluent speakers of Filipino and English and VCFP, AMLA, and MMD in all three languages. We checked for linguistic and conceptual equivalence in the translated documents. We de-identified all participants and assigned pseudonyms. We thematically analyzed the data to identify national and local policies on self-care using Microsoft Excel (Microsoft). The quotes presented in this paper are either in the original English or translated from Filipino or Cebuano.

**Policy review**

***Eligibility criteria***

Records were included in the study if they contained any of the keywords in the context of general health and/or the following health conditions: back pain, allergic rhinitis, general acute pain, cough, cold, diarrhea, constipation, and stress. The search was limited to documents published from January 1, 2010 to January 31, 2022. Pending bills and previously filed bills in Congress have been included in this review because these form part of the legislative agenda on health and have the potential of being passed into law.

***Information sources***

We conducted the review of policies in the following local databases from January to March 2022: DOH Document Management and Archiving System (DMAS), House of Representatives Legislative Information System (LEGIS), and Senate Legislative Documents. This review followed the general steps of Joanna Briggs Methodology (Peters *et al.*, 2020; Antonio *et al.*, 2021). We were interested in self-care in the context of self-limiting conditions including back pain, allergic rhinitis, general acute pain, cough, cold, diarrhea, constipation, and stress in the Philippine setting. Of note is that these databases do not have advanced search features, requiring searching using a toolbar with no restrictions.

***Search strategy***

To generate the initial list of records, the following initial keywords were: self-care, self-medication, over-the-counter, OTC, OTC switch, nonprescription drugs, self-efficacy, self-management, prescription sharing, TCAM, complementary therapies, traditional medicine, traditional therapy, complementary therapies, complementary and alternative medicine, herbal medicine, herbal therapy, medicinal herbs, herbal extract, herbal product, herbal supplement. These keywords were informed by our qualitative study and a preliminary search of published literature. Our initial search yielded no results or a large volume of irrelevant results. We therefore narrowed our keywords to: health promotion, over-the-counter medicines, and traditional and complementary medicine. These keywords were the most used in the databases and yielded results relevant to our study.

***Selection of sources***

Duplicate records of bills were removed. For legislative documents, duplicates include previous versions of bills, bills with similar titles, and bills carrying similar proposals. All unique documents that met our criteria were then retrieved and subjected to full-text review. The analysis was conducted to ascertain that the policy documents included pertained to and/or were relevant to self-care. Two of the authors performed the screening and analysis, and any disagreements were resolved either by consensus or through a third party.

***Data extraction and synthesis***

We summarized and thematically analyzed the policy documents to identify key themes and propose recommendations to promote self-care in the country. We used Covidence in our screening and extraction. The following information were extracted: the classification and source of the policy document, its title/subject, the year it was institutionalized, and a short summary of the document pertaining to self-care.

***Process of triangulation***

We identified policies on self-care from our interviews and focus group discussions, triangulated by our policy review conducted using a scoping method approach. All policies identified, regardless if the documents mentioned the term ‘self-care’ or its other aspects and more commonly used synonyms (e.g., self-medication, self-management, etc.), were reviewed for their relevance to the scope and objectives of the study. All policies that were within the scope of self-care on the health conditions of interest were included and thematically analyzed.

**Appendix B. Limitations**

Our study focused on self-care policies for common acute conditions as chronic conditions will demand a discourse with greater focus on rehabilitation, treatment adherence, and palliative care (Hearn *et al.*, 2019). Previous studies on self-care have also generally focused on chronic conditions, reflected in the proposed self-care research agenda (Riegel *et al.*, 2021), which highlights a gap on the evidence concerning self-care for acute conditions. However, in doing this case study and in-depth analysis of policies, we highlighted the importance of health promotion and health literacy, which are important regardless of the disease and condition.

One implementation issue that our study encountered was the lack of a search engine for the local databases, as well as the lack of standardized keywords and poor tagging of policy documents. This could have resulted in the exclusion of important policy documents in the review process. However, we addressed this by complementing our search with data from our focus group discussions and key informant interviews.

**References**

Antonio CAT, Amit AML, Reyes MSGL, et al. 2021. Practical guidance in the conduct of a

scoping review: insights from experience in the College of Public Health, University of the Philippines Manila. Acta Medica Philippina 55(7): 775-780.

Hearn J, Ssinabulya I, Schwartz JI, et al. 2019. Self-management of non-communicable

diseases in low- and middle-income countries: a scoping review. PLOS ONE 14: e0219141.

Microsoft. Microsoft Excel: Spreadsheet Software Microsoft; Available from:

https://www.microsoft.com/en-ww/microsoft-365/excel.

Peters M, Godfrey C, McInerney P, et al. 2020. Chapter 11: Scoping reviews. In: Aromataris E,

Munn Z (eds). JBI Manual for Evidence Synthesis. JBI: Adelaide.

Riegel B, Dunbar SB, Fitzsimons D, et al. 2021. Self-care research: Where are we now? Where

are we going? International Journal of Nursing Studies 116: 103402.
